# Awareness and attitude toward using dental magnification among dental students and residents at King Abdulaziz University, Faculty of Dentistry

**DOI:** 10.1186/s12903-016-0254-4

**Published:** 2016-07-19

**Authors:** Turki Y. Alhazzazi, Nouran A. Alzebiani, Samaher K. Alotaibi, Dania F. Bogari, Ghaida T. Bakalka, Loai W. Hazzazi, Ahmed M. Jan, Neville J. McDonald

**Affiliations:** Department of Oral Biology, King Abdulaziz University, Faculty of Dentistry, Building 14, P.O. Box 80200, 21589 Jeddah, Saudi Arabia; Department of Endodontics, King Abdulaziz University, Faculty of Dentistry, Jeddah, Saudi Arabia; King Abdulaziz Medical City, National Guard Hospital, Jeddah, Saudi Arabia; Department of Oral and Maxillofacial Surgery, King Abdulaziz University, Faculty of Dentistry, Jeddah, Saudi Arabia; Cariology, Restorative Sciences, and Endodontics Department, University of Michigan, Ann Arbor, Michigan USA

**Keywords:** Magnification, KAUFD, Awareness, Dental students, Dentistry

## Abstract

**Background:**

The authors conducted a study aimed to assess the awareness and attitude among dental students and residents at King Abdulaziz University, Faculty of Dentistry (KAUFD) toward using dental magnification.

**Methods:**

An e-questionnaire was formulated then sent to dental students and residents (*n* = 651). The questionnaire included questions that assessed both the awareness and attitude toward using dental magnification. Data were analyzed using IBM SPSS version 22. The chi-square test was used to establish relationships between categorical variables.

**Results:**

The response rate was 69.7 % (*n* = 454). Of those, 78.1 % did not use magnification during dental procedures. However, 81.8 % agreed that dental magnification could enhance the accuracy and quality of their dental work. Thus, 91.6 % thought it would be useful in endodontics and 46.3 % voted for surgery. Of the 21.9 % that used magnification, dental loupes were mostly used, 55.9 %. The majority (59.4 %) of the participants believed that using dental magnification should be introduced by faculty beginning in Year I of dental school.

**Conclusions:**

Among our respondents, most of the undergraduate students did not use dental magnification nor attended courses in the use of dental magnifications. However, most of the students were aware of its significance in improving the accuracy and quality of their work.

## Background

Magnification in general is considered one of the great revolutions in science, and specifically in dentistry. This revolution allowed scientists to undertake precise studies in the natural world and accelerate progress in the fields of medicine and dentistry.

The idea of enhanced magnification was introduced to dentistry in the form of an operating microscope in the late 1970s. The Dental Operating Microscope (DOM) was introduced to endodontics in the late 1980s by Dr. Gary Carr. He established that magnification and illumination could be helpful in increasing the success rate of endodontic apical surgeries [1]. Regular use of a DOM in various dental specialties was advocated for caries diagnosis and excavation [2], crown margins and post placement [3], surgical incisions, bone and soft tissue grafting procedures [4], and furcation and perforation repairs [2].

Despite the significant cost and relatively prolonged learning curve when starting using magnification tools in the different dental specialties, it is generally, believed that their use should be advocated. They are becoming an integral part of the era of modern microsurgery in various fields of dentistry. Advantages include improving the quality of treatment, achieving better posture during dental practice, reducing visual stress, and decreasing musculoskeletal injury when implementing them in our dental practices. These advantages occur only after going through the proper training and after gaining the required skills [5–9].

The clear advantage of using a DOM in dentistry, resulted in the Commission on Dental Accreditation requiring training in microscopy in all Advanced Endodontic Programs, as of 01 July 1997 [10]. In 2012, the American Association of Endodontics (AAE) stated that endodontics could benefit from the use of a DOM in the following procedures: detecting hidden canals, especially the MB2 canal in maxillary molars, retreatment cases where removing materials such as obturation materials and posts in necessary, removing broken instruments, achieving proper conservative access through superior visualization of the pulp chamber and canal orifices, perforation repairs, and all steps of endodontic surgery [11].

A study conducted by Buhrley et al. in 2002 [12], concluded that using a microscope or at least dental loupes is essential in locating the second mesiobuccal canal (MB2). Thus, the frequency of detecting MB2 for the microscope, dental loupes, and no magnification groups was 71.1, 62.5, and 17.2 %, respectively. This shows that the accuracy while using magnification to detect MB2 is about three fold more than the non-magnification group. This also emphasizes the importance of using magnification for locating the MB2 canals during endodontic treatment in order to provide better treatment outcome [12].

Tsesis et al. in 2006 [13] found that modern surgical endodontic treatment using microscope and ultrasonic tips resulted in significantly better therapeutic outcome compared to traditional techniques that did not use microscope and used burs for retrograde cavities preparation (91.1 % vs 44.2 %, *p* < 0.0001) [13]. This further supports the use of modern technology, including microscopy, as a standard of care in modern surgical endodontic treatment. Nevertheless, only 9 % of general dental practitioners routinely used dental magnification in one Scottish study [14]. Similarly, another study reported an increase in the use of dental loupes among general dental practitioners (44 %). However, a lower percentage was reported for graduated dental students (28 %) [15]. This underscores the importance of increasing the awareness of using dental magnification in our profession, and emphasizing the use of dental magnification early in dental schools.

Limited numbers of studies have been published on the use of dental magnification among dental students and residents. Maggio et al. conducted a study on the effect of magnification loupes on the performance of preclinical dental students. Students using magnification loupes worked faster, completed more teeth preparations, used computer-assisted evaluation less frequently, and demonstrated better overall performances [16]. Similarly, a recently conducted study on dental interns and final year undergraduate students, showed statistically significant improvement in the outcome of class II cavity preparations for those using magnification loupes [17].

Thus, the aim of this study was to assess the awareness and attitude, among dental students and residents at King Abdulaziz University, Faculty of Dentistry (KAUFD), toward using dental magnification.

## Methods

An e-questionnaire was formulated and sent via email to dental students and residents (postgraduate students) at KAUFD (*n* = 651). Postgraduate students included in this study were from the advanced restorative and the advanced endodontics programs. The sent email stem contained the title and the purpose of the research project, and clearly explained the usefulness of their agreeing to participate in the research project. All students were at least 18 years of age. The questionnaire included questions that assessed both the awareness and attitude toward using dental magnification among the group sample. The e-questionnaire constituted written consent that was approved by the research ethical committee board from KAUFD. This project was approved by the committee, and was in full accordance with the World Medical Association Declaration of Helsinki.

### Statistical methodology

Data were analyzed using IBM SPSS version 22. Simple descriptive statistics were used to define characteristics of the variables using numbers and percentages for categorical variables. To establish relationships between categorical variables, the chi-square test was used, and statistical significance was recognized when *p* <0.05.

## Results

Four hundred fifty four questionnaires were answered with an overall response rate of 69.7 %. The response rates were 59.5 and 40.5 % for females and males, respectively. Levels of education of the respondents were sixth year and fourth year undergraduate students, 24.0 and 23.6 %, respectively. Unfortunately, only 3.5 % of the respondents were postgraduate students (ten students were from the advanced restorative program, and 6 students were from the advanced endodontics program) (Table 1).

**Table 1:** Demographic data of the participants (Gender and level of education)

Demographics		Count	Percent
Total		454	100.0
Gender	Male	184	40.5
	Female	270	59.5
Which level of education are you currently at in the dental school?	Third Year	103	22.7
	Fourth Year	107	23.6
	Fifth Year	94	20.7
	Sixth Year	109	24.0
	Internship	25	5.5
	Saudi Board	16	3.5
	Resident		
Which level of education are you currently at in the dental school?	Under Grad	438	96.5
	Post Grad	16	3.5

**Fig. 1:**
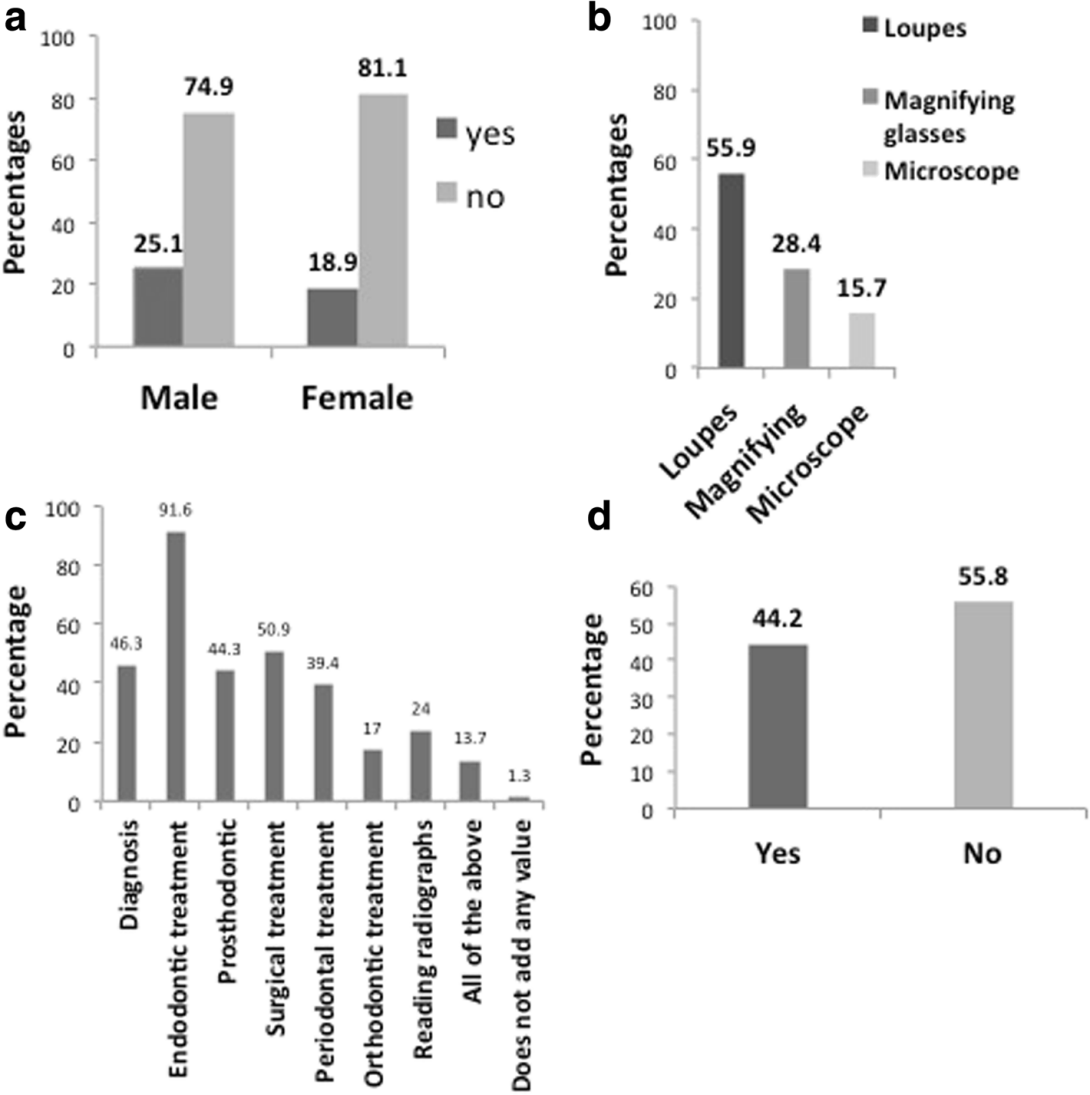
Frequency and opinion of students toward dental magnification usage during dental procedures. **a** Gender difference among magnification users. **b** Types of dental magnification used among our participants. **c** Opinion toward the usefulness use of dental magnification depending on dental specialties. **d** Opinion toward relying on dental magnification while practicing dentistry

Only 21.4 % of the participants used dental magnification during any dental treatment (Table 2). Our results showed that males (25.1 %) used dental magnification more often than females (18.9 %). However, this difference was not statistically significant (*p > 0.05*) (Fig. 1a).

**Table 2:** Overall attitude and awareness of student toward using dental magnification

Variables		Count	Percent
A) Do you use magnification during dental process?	Yes	97	21.4
	No	356	78.6
	Total	453	100.0
B) If you didn’t have your magnifying tool on you today, and you are required to work on a patient, what would you do?	Still work on my patient	127	55.7
	I will reschedule the patient because I don’t feel comfortable	23	10.1
	Depend on the procedure, I may or may not work on the patient.	78	34.2
	Total	228	100.0
C) Do you believe that dental magnification can enhance the accuracy and quality of your work?	Yes	368	81.8
	No	82	18.2
	Total	450	100.0

**Fig. 2:**
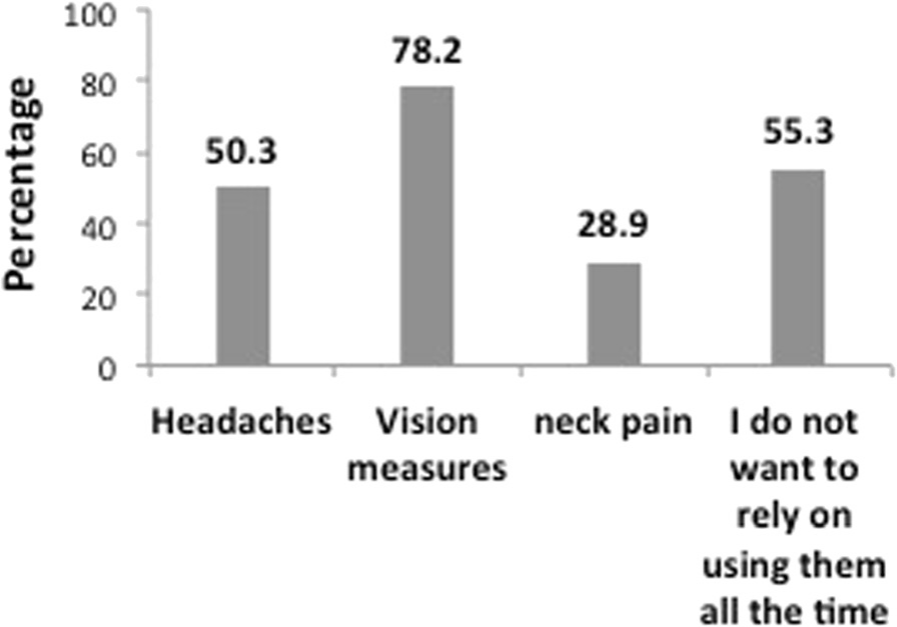
Reasons of not preferring using dental magnification while practicing dentistry

There was a significant difference between undergraduate and postgraduate dental students using dental magnification (19.7 % vs. 68.8 %, *p* <0.001) (Table 3). In addition, there was a statistically significant difference between undergraduate and postgraduate students in attending courses on the use of dental magnification (47.3 % vs. 100 %, *p* <0.001). Loupes were the most widely used form of magnification (55.9 %) followed by magnifying glasses (28.4 %) (Fig. 1b). Most of the participants believed that, lacking magnifying tools was not important, and the majority would resume treating their patients without it (Table 2). Although, 81.8 % agreed that dental magnification can enhance the accuracy and quality of their work (Table 2). When it comes to dental specialties, 91.6 % believed it would be useful in endodontics and 50.9 % voted for surgical treatment (Fig. 1c). Interestingly, 44.2 % (Fig. 1d) of students believed that relying on magnification while performing dental work can be a problem. They thought it will influence their vision measures, make them rely on using them all the time, and others suffered from headaches (Fig. 2).

**Table 3:** Undergraduate vs Postgraduate students attitude toward using dental magnification and attending dental magnification courses

Variables		Total	Which level of education are you currently at in the dental school?		*p*-value^a^
			Under grad	Post grad	
Total		453	437 (96.5 %)	16 (3.5 %)	N/A
A) Do you use magnification during dental process?	Yes	97	86 (19.7 %)	11 (68.8 %)	<0.001
	No	356	351 (80.3 %)	5 (31.3 %)	
B) Have you ever attended courses or classes on the use of magnification in the dental field?	Yes	221	205 (47.3 %)	16 (100.0 %)	<0.001
	No	228	228 (52.7 %)	0 (0.0 %)	

^a^-significant using Chi-Square test @ <0.05 level

**Fig. 3:**
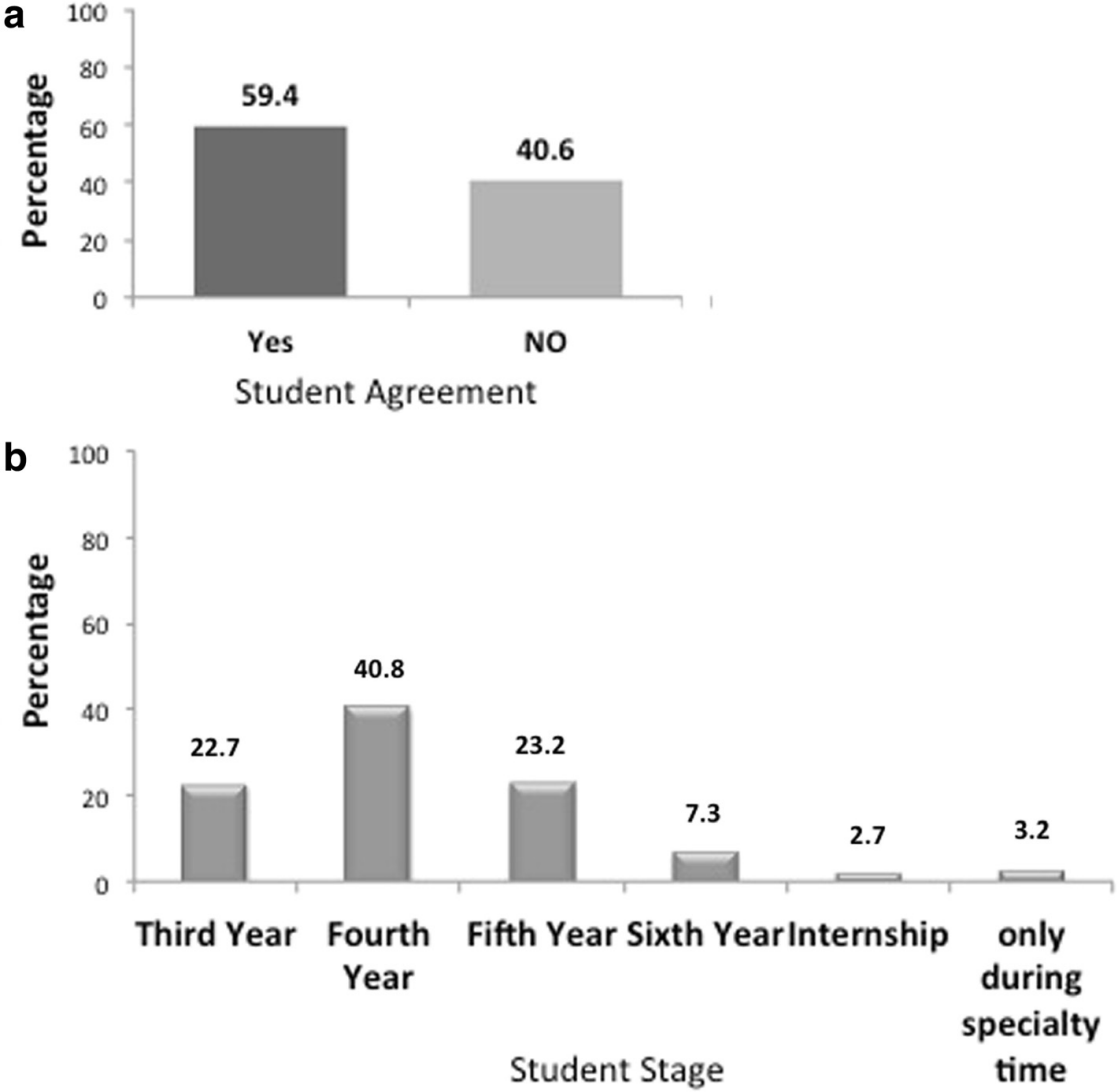
Students opinion toward faculty reinforcement of using dental magnification early during dental school: **a** Percentage of supporting students. **b** Stage of when it should be reinforced

The majority of the students (59.4 %) believed that, using dental magnification should be reinforced by faculty earlier during dental school (Fig. 3a). They voted for fourth year as the right time to introduce this concept (Fig. 3b). About 78.1 % of our students agreed that less than 25 % of the teaching faculty at the dental school used dental magnification.

## Discussion

Limited numbers of studies have been published on the use of dental magnification among dental students and residents especially in the kingdom of Saudi Arabia. The overall routine use of magnification among dental students and residents at KAUFD was less than we had anticipated.

Unlike the Scotland study [14], our study revealed a significant difference between undergraduate and postgraduate students using dental magnification (19.7 % vs 68.8 %, *p* <0.001). This may be due to the attended courses by postgraduate students on the use of dental magnification compared to the undergraduate students. Our participants agreed that dental magnification would be most useful in endodontics followed by surgical procedures, while the Scottish study found it most useful in crown and bridge procedures followed by diagnosis [14].

A study conducted in the University of Newcastle, reported that the biggest drawbacks of wearing loupes among dental hygienists. Drawbacks included the need of adjustment period, limited depth of vision, headache, dizziness and trouble in infection control measures [18]. While according to our data, 44.2 % of the participants believed that relying on dental magnification while performing dental work could be a problem. The majority assumed that it would affect their vision. This is a misconception according to Dr. Christensen [6], thus, using magnifying loupes does not weaken or affect the eye. Hence, after a period of using magnification, the users would become familiarized to see more detail than with the naked eye. Furthermore, the uncomfortable feeling after stopping the use of magnifying devices is simply due to the fact that the eyes need time to readjust to the normal vision [6].

There is a high variation in the near vision acuity between individuals and it is believed that the vision acuity declines throughout individual’s lifetime. Using magnification tools can significantly enhance the vision acuity independent of age or natural vision [19]. In addition, several studies suggest that regular optometric check up for the dentist every two years, especially after the age of 40 is essential to overcome presbyopia [20, 21].

The musculoskeletal health of dentists has been subject of argument worldwide. All the dentists could face occupational musculoskeletal hazard [8, 22]. Some studies report that every one out of ten dentist have poor general health, and every three out of ten dentists have poor physical state [23]. These problems can be prevented by increasing awareness toward ergonomics during dental practices [24, 25]. Using the suitable visual magnification should be considered for all dentists for more precise, convenient, and pleasant dental performance. This may decrease the risk of musculoskeletal injury [6, 8]. In addition, implementing the concept of using magnification loupes early in dental education programs can significantly improve students posture during dental work [9]. Unfortunately, in KAUFD, the use of dental magnification tools is not formally taught. However, this concept is advocated during continuing educational courses throughout the academic year.

The majority of the participants believed that using dental magnification should be reinforced by faculty early during dental school. In addition, they believed that fourth year is the right time to introduce this concept. Our school awards bachelor in dental medicine and surgery (BDS) in seven years of studies. However, it is not until year four, when students start treating patients. This may explain the reason why students seem to believe that fourth year would be the right time to implement the concept of using dental magnifications. Nevertheless, BDS students start their preclinical studies in operative dentistry in third year. Thus, third year might also be an attractive year to introduce dental magnification to students to provide a smooth learning curve before the students begin treating patients. This is in agreement with many dental schools worldwide that are now actively encouraging their undergraduates to use dental magnification during undergraduate dental education [26]. One of the future directions of this study is to investigate misconceptions among undergraduate and postgraduate students toward the use of dental magnification.

## Conclusions

In KAUFD, most of the undergraduate students had not used dental magnification and never attended courses in its use. Most of the students were aware of dental magnification significance in improving the accuracy and quality of their work. Therefore, reinforcement is needed for both students and faculty to use dental magnification during dental procedures. The outcome of this study would be of help to KAUFD stakeholders and academic curriculum developers while designing new policies and revising the current curriculum. In addition, it provides evidence to improve standards of care at KAUFD by adding dental magnification as an integral armamentarium of undergraduate dental education.

## Abbreviations

AAE, American Association of Endodontics; BDS, bachelor in dental medicine and surgery; DOM, dental operating microscope; KAUFD, King Abdulaziz University, Faculty of Dentistry; MB2, second mesiobuccal canal
